# *Cajetanus Tacconus* de Morbo, qui lapsum ab excelso loco, et inde ortum terrorem consecutus est

**Published:** 1785

**Authors:** 


### SECTION II.

### BOOKS.

I. Cajetanus Tacconus *de Morbo, qui lapſum ab excelſo loco, et inde ortum terrorem conſecutus eſt.* Vide *De Bononienſi Scientiarum et Artium Inſtituto atque Academia Commentariorum Tomum Sextum.* 4to. Bononiæ, 1783.

THE patient, whoſe caſe is here related, was a poor girl about fifteen years old, who ſubſiſted by begging alms in the neighbourhood of the author, and who attracted his attention by the ſingularity of her appearance. She walked ſlowly, and ſeemingly with great difficulty, and uſed to ſtop a little at every third ſtep. He obſerved, likewiſe, that her hands, arms, face, lips, and even the tunica conjunctiva of her eyes, were of a livid colour. Theſe appearances excited his curioſity to learn the particulars of her hiſtory.

She informed him that ſhe felt a conſtant pulſation in the left ſide of her cheſt, and extreme weakneſs in all her limbs; that ſhe was able to ſwallow only very thin or liquid aliment, and even that not without much difficulty; culty ; that ſhe was generally coſtive ; and that her complaints, ſhe had obſerved, were conſtantly much increaſed during the winter. She had as yet had no appearance of the catamenia ; her voice was low and faultering ; and ſhe had a quick, weak pulſe. — Her diſorder, ſhe ſaid, firſt took place when ſhe was about five years old, and was attributed to a ſudden and exceſſive fright ſhe had experienced at that time in conſequence of a fall from a great height.

During the ſpace of three years from the time our author firſt ſpoke to her, hardly a day paſſed without his ſeeing or converſing with her ; and yet, during the whole of this period, though he obſerved her with the greateſt attention, he could never diſcover that her noſtrils, thorax, or abdomen, had the leaſt degree of motion in reſpiration. In the mean time her complaints became gradually more diſtreſſing ; and at length, during the winter ſeaſon, when the weather was unuſually cold and wet, ſhe began to complain of a pain in her left ſide, and to diſcharge by the mouth a black grumous blood. Still, however, ſhe continued to place herſelf in the ſtreet as uſual, till the four-and-twentieth day after the laſt-mentioned ſymptoms had made their appearance, when her ſtrength

ſtrength was ſo much reduced, that ſhe was obliged to be carried to her lodging, which was hard by, and there ſhe ſoon after died.

On examination of the dead body, our author found that the lividity which he had formerly obſerved in the face, neck, and hands, was general over the whole body; the ſtomach, liver, and omentum, appeared to be larger than uſual; the diaphragm was extremely thin, and ſo much preſſed upwards by the inteſtines, that the capacity of the thorax was extremely ſmall; the lungs were dry, hard, and contracted; the left lobe was of a dark livid colour, and adhered to the pleura at the part where the patient had complained of pain. About three ounces of black, grumous blood were found on the ſurface of the diaphragm, and in the trachea; but the moſt remarkable appearance was in the ſtructure of the heart, which was ſo diſtorted as to approach rather to a cubical than a conical ſhape. With reſpect to its two ventricles, it was obſerved, that the left ventricle, in its ſhape and ſtructure, appeared as the right ventricle uſually does in a natural ſtate, and *vice verſa*. The pulmonary artery, by the adheſion of the ſigmoid valves to each other, was ſo far cloſed that water could be preſſed

 into into it only through a very ſmall opening, and this our author ſuſpected to have been made by a probe with which he had examined the ſtate of the valves. The pulmonary vein was empty and contracted. The canalis arterioſus was cloſed; but the foramen ovale was found open, and larger than it uſually is in the fœtus. The remainder of the diſſection afforded nothing preternatural.

After deſcribing the appearances that occurred in the diſſection, our author proceeds to offer an explanation of them. The opening of the foramen ovale he attributes to the fall and conſequent terror. From the time the blood began to circulate again through this channel, the pulmonary artery, he obſerves, muſt have become gradually leſs pervious, and the blood, by being no longer expoſed to the action of the air in the lungs, would ſoon ceaſe to be of a florid colour. This change in the circulation, he thinks, is ſufficient to account for the gradual alteration in the figure of the heart; and in proportion as the left ventricle became more diſtended than the right, he ſuppoſes that, like an aneuriſm, it might occaſion the pulſation of which the patient complained in the left ſide of her cheſt.—Such is the author's explanation of thefe extraordinary appearances; but the reader who has perufed the cafes of mal-conformation of the heart, lately publifhed* in this country, will, perhaps, be inclined to attribute the ftate of that organ, in the prefent inftance, to an original defect of ftructure, rather than to any accidental change in the courfe of the circulation after birth. But whatever might be the caufe of the appearances in queftion, the facts here related are certainly extremely curious, and may ferve as additional proofs of the deviation from the natural conformation of the heart, under which life, to a certain period at leaft, may be carried on.

---

II. Chriftianus Fridericus Reufs *de virtutibus Caricis arenariæ radicis Sarfaparillæ vires fere fuperantibus.* Vide *Novorum actorum phyfico-medicorum Academiæ Cæfareæ Leopoldino-Carolinæ Naturæ Curioforum Tomum feptimum.* 4to. Norimbergæ, 1783.

THE defign of this paper is to recommend the root of the *Carex arenaria* as a fub-

* See Medical Tranfactions, Vol. III. p. 339; and Medical Obfervations and Inquiries, Vol. VI. p. 291.

